# Adeno-associated virus-mediated gene transfer of arginine decarboxylase to the central nervous system prevents opioid analgesic tolerance

**DOI:** 10.3389/fpain.2023.1269017

**Published:** 2024-02-09

**Authors:** Caroline C. Churchill, Cristina D. Peterson, Kelley F. Kitto, Kelsey R. Pflepsen, Lalitha R. Belur, R. Scott McIvor, Lucy Vulchanova, George L. Wilcox, Carolyn A. Fairbanks

**Affiliations:** ^1^ Department of Experimental and Clinical Pharmacology, University of Minnesota, Minneapolis, MN, United States; ^2^ Department of Neuroscience, University of Minnesota, Minneapolis, MN, United States; ^3^ Department of Pharmaceutics, University of Minnesota, Minneapolis, MN, United States; ^4^ Department of Genetics Cell Biology and Development, University of Minnesota, Minneapolis, MN, United States; ^5^ Department of Pharmacology, University of Minnesota, Minneapolis, MN, United States; ^6^ Department of Dermatology, University of Minnesota, Minneapolis, MN, United States

**Keywords:** AAV5, agmatine, human arginine decarboxylase, intrathecal, opioid tolerance, AAV9, NMDA receptor antagonism

## Abstract

Agmatine, a decarboxylated form of L-arginine, prevents opioid analgesic tolerance, dependence, and self-administration when given by both central and systemic routes of administration. Endogenous agmatine has been previously detected in the central nervous system. The presence of a biochemical pathway for agmatine synthesis offers the opportunity for site-specific overexpression of the presumptive synthetic enzyme for local therapeutic effects. In the present study, we evaluated the development of opioid analgesic tolerance in ICR-CD1 mice pre-treated with either vehicle control or intrathecally delivered adeno-associated viral vectors (AAV) carrying the gene for human arginine decarboxylase (hADC). Vehicle-treated or AAV-hADC-treated mice were each further divided into two groups which received repeated delivery over three days of either saline or systemically-delivered morphine intended to induce opioid analgesic tolerance. Morphine analgesic dose-response curves were constructed in all subjects on day four using the warm water tail flick assay as the dependent measure. We observed that pre-treatment with AAV-hADC prevented the development of analgesic tolerance to morphine. Peripheral and central nervous system tissues were collected and analyzed for presence of hADC mRNA. In a similar experiment, AAV-hADC pre-treatment prevented the development of analgesic tolerance to a high dose of the opioid neuropeptide endomorphin-2. Intrathecal delivery of anti-agmatine IgG (but not normal IgG) reversed the inhibition of endomorphin-2 analgesic tolerance in AAV-hADC-treated mice. To summarize, we report here the effects of AAV-mediated gene transfer of human ADC (hADC) in models of opioid-induced analgesic tolerance. This study suggests that gene therapy may contribute to reducing opioid analgesic tolerance.

## 1 Introduction

Opioid pharmacotherapy remains an important option for individuals with chronic pain arising from critical illnesses that are not alleviated by non-opioid or non-pharmacological pain management approaches (1, 2), particularly for malignant chronic pain. However, a panel of complex side effects accompanies long-term opioid pharmacotherapy, including, but not limited to, the development of opioid analgesic tolerance (1, 2). The development of analgesic tolerance for patients on chronic opioid therapy can result in significant dose escalation (3) resulting in increased adverse side effects (4), a need for more invasive delivery approaches, or the inability to adequately control pain [ceiling effect (5)]. Multiple strategies are used with varying success for these patients, such as opioid rotation, drug holiday, or inclusion of adjuvant analgesics such as ketamine (5). The application of ketamine as an analgesic adjuvant strategy (1, 2) to reduce the induction of opioid analgesic tolerance is based on the well-established observation that antagonism of NMDA receptors reduces the development of opioid analgesic tolerance (6). More than thirty years of pharmacological studies have shown that antagonism of the NMDA receptor (6–10) or inhibition of the nitric oxide synthase (NOS) enzyme (10–12) inhibits the induction of opioid-induced analgesic tolerance.

Agmatine was discovered in the central nervous system (CNS) in 1994 (13), closely followed by the cloning of its synthetic (14) and degradative enzyme systems (15) that are also expressed in CNS. Subsequently, neuropharmacological investigations have converged on an inhibitory role for agmatine in multiple models of behavioral neural plasticity (16). We and others have shown that agmatine inhibits the induction of opioid analgesic tolerance when given either systemically (17) or centrally (18–20). Evidence suggests that agmatine antagonizes the NMDA receptor (21, 22) and/or inhibits the NOS enzyme (21, 23–25) to exert its inhibition of opioid analgesic tolerence.

Since agmatine is a naturally occurring product with a presumptive synthetic enzyme that is expressed in the central nervous system (26), an opportunity arises to design an agmatine-based gene therapy with site-directed expression within the CNS. Toward this end, we developed an adeno-associated viral vector (AAV) that carries the presumptive synthetic gene for agmatine (27). We have demonstrated that AAV viral vectors delivered by intrathecal injection distribute through the central nervous system (28) and provide robust gene transfer to sensory neurons at all levels of the neuraxis (29). We have also shown that intrathecal delivery of an AAV vector containing the gene for human arginine decarboxylase (AAV-hADC) leads to persistent alleviation of neuropathic pain in rodents (27).

Since opioid analgesic tolerance and neuropathic pain and are both dependent on NMDA receptor activation (30) and agmatine reduces both neuropathic pain (31, 32) and opioid analgesic tolerance (18–20), we hypothesized that intrathecal delivery of AAV-hADC would reduce the induction of analgesic tolerance to opioids. To test this hypothesis, the development of chronic systemic morphine analgesic tolerance or acute intrathecal endomorphin-2 analgesic tolerance was compared between AAV-hADC-treated mice and vehicle-injected controls.

## 2 Materials and methods

### 2.1 Research subjects

All subjects were 20–25 g adult male ICR-CD1 mice (Envigo, Madison, WI). All experiments were approved by the University of Minnesota Institutional Animal Care and Use Committee.

### 2.2 Chemicals

Morphine sulfate was a gift from the National Institute on Drug Abuse. Endomorphin-2 (ENDO-2, YPFF) was synthesized by the University of Minnesota's microchemical facility, Minneapolis, MN. Agmatine sulfate and NMDA were obtained from Sigma Chemical (St. Louis, MO) and dissolved in 0.9% NaCl.

### 2.3 AAV vectors

The development of the human ADC-containing vector was previously described (27). The vector titer was 8.24 × 10^12^_ _vector genomes/ml (vg/ml) for AAV5-hADC and 6.37 × 10^12^ vector genomes/ml (vg/ml) for AAV9-hADC which was diluted 1:3 in saline for a final injected titer of 2.1 × 10^12^ vector genomes/ml (vg/ml).

### 2.4 Gene expression by RT-PCR

#### 2.4.1 PCR method used in AAV5-hADC experiments

Total RNA extraction and qualitative PCR has been previously described by Peterson and colleagues (27) and was based upon the method reported by Seybold and colleagues (33). The sequences of the primers used are as follows: AAV5 experiments: hADC forward primer: GCCTTGGACCTGTACTTCCC; hADC reverse primer: CTGGTCCGTGGATGGTTTCT.

#### 2.4.2 PCR method used in AAV9-hADC experiments

Total RNA was extracted from tissue using RNAzol RT. *Reverse-transcription (RT)*. Four hundred micrograms RNA was reverse-transcribed in RT master mixture (final concentrations: 2.5 mM MgCl2, 1× PCR Buffer II, 10 mM dithiothreitol, 1.25 μM random hexamers, 50 U RNase inhibitor, 250 µM dNTP and 75 U MMLV reverse transcriptase; Applied Biosystems/Life Technologies, Grand Island, NY) in a volume of 20 µl. The reactions were incubated at 25 °C for 10 min followed by 42 °C for 20 min followed by 65 °C for 3 min, cooled on ice and stored at −20 °C until used for PCR. Two microliters of RT product is added to PCR master mixture (final concentrations: 1× Lightcycler 480 SyBr green mastermix and 300 nM of each primer in a final volume of 20 µl and pipetted into a well of a 96-well plate (hADC forward primer: GTACCCGAGACCTGCTGAAG; hADC reverse primer: GGACCAACTCCATCTCTGCC). The plate is then inserted into a Lightcycler 480 II (Roche) and a PCR program was run. The program consisted of one cycle of 95°C for 8 min (polymerase activation) followed by 55 cycles of: 95 °C for 15 s, 60 °C for 15 s and 72 °C for 15 s. Following amplification, the built-in thermal melting (Tm) and second derivative maximum (to determine quantification cycle, Cq) programs were also run. The PCR products and data were analyzed using 1.5% agarose gel electrophoresis, Lightcycler 480 software v.1.5, Photoshop CS v.5.1 and GraphPad Prism v6f software.

### 2.5 Thermal nociception assay

Thermal nociception was assessed with the warm water (52.5°C) tail immersion assay (18, 20, 34). The time that lapses between the mouse tail entering the water to the mouse flicking its tail is the dependent measure. Initial tail-flick latencies were taken on each individual mice prior to treatment (for a sample of *n* = 42, mean = 3.32 s, and S.D. = 0.65 s). Each subject's baseline value was used as an internal control. The maximum possible effect (%MPE) was calculated accordingly: %MPE = (postdrug latency − predrug latency)/(cutoff − predrug latency) × 100%. A maximum score of 100% was given mice that do not flick their tails prior to a cutoff of 12 s. Twelve seconds is the maximum time for the tail to be allowed to remain in the water at that temperature in order to prevent thermal injury.

### 2.6 Rotarod assay

After two training sessions, mice were permitted to walk for a maximum of 300 s on an accelerating (4–40 rpm) rotarod (Ugo Basile, Varese, Italy). The dependent measure was the time spent remaining on the rotarod. We statistically compared the mean time that each subject remained on the rotarod across the four treatment groups using a one-way ANOVA with Tukey's *post hoc* test for comparisons between multiple groups.

### 2.7 Dose-response analysis

At least 3–4 doses were included in the development of each dose-response curve for each experimental group. The ED_50_ values and 95% confidence intervals (CIs) of drugs were calculated by parametric linear regression analysis of the log dose–response curves. These methods have been previously described (35). These calculations were performed using FlashCalc, a pharmacological statistics program written by Dr. Michael Ossipov, University of Arizona, Tucson, AZ.

### 2.8 Intrathecal injections

The AAV-hADC viral vectors or vehicle (0.9% normal saline) were given intrathecally (i.t.) by lumbar puncture in awake mice as previously described (36, 37). In order to conserve the AAV5-hADC vector we modified the delivery method (38). The needle (30-gauge, 0.5-inch) was connected to a length of PE10 tubing, which was itself connected to a second needle (a 30-gauge, 0.5-inch) that was fitted to a 50-µl Luer-hub Hamilton syringe. For both AAV-hADC experiments, 10 µl of the injectate which was comprised of 8.24 × 10^12^ viral vector genomes/ml (AAV5) or 2.1 × 10^12^ vector genomes/ml viral vector genomes (AAV9) were injected intrathecally. The injection was performed by gently grasping the hip bones of the rodent and inserting the needle (bevel side up) at about a 45° angle centered on the iliac crest. The penetration of the dura is accompanied by a reflexive flick of the tail. After intathecal delivery, the mice were returned to the animal housing area where they stayed until the the opioid analgesic tolerance experiment was initiated. Extraction of spinal cord, fourth ventricle choroid plexus, midbrain (PAG), and DRG for RT-PCR took place on the last day of the experiment after all analgesic testing had been completed.

### 2.9 Chronic morphine analgesic tolerance induction

Baseline tail flick latencies were taken on all mice prior to drug exposure. Mice acquired analgesic tolerance to morphine following repeated administration of subcutaneously delivered morphine over the course of three days. A single injection of morphine was delivered on day 1 (3 mg/kg) followed by three injections daily at 9 am, 5 pm, and 11 pm on Days 2 (3 mg/kg) and 3 (5 mg/kg). A total of seven subcutaneous injections were delivered over the course of the induction schedule. A cohort of control mice received an equivalent number of subcutaneous saline injections at the same time as the morphine treatment groups. All injections were given in a volume of 100 µl/10 grams. Cumulative dose-response curves of morphine were then constructed in both the morphine pre-treatment and saline pre-treatment groups in the following way: a single dose of morphine was injected, and the tail flick latency was assessed at 30 min after injection. A second dose of morphine was delivered and the process repeated. Doses were delivered in this manner until a complete dose-response curve was constructed in each treatment group.

### 2.10 Acute endomorphin-2 analgesic tolerance induction

Baseline tail flick latencies were assessed for all mice prior to drug exposure. Mice developed analgesic tolerance to endomorphin-2 (ENDO-2) following a single intrathecal injection of a large dose (10 nmol, 5 µl) of ENDO-2. A cohort of control mice were injected with an equivalent volume of intrathecal saline (5 µl) at the same time as the ENDO-2-treatment groups. Thirty minutes later, tail flick latencies were measured to verify that the responses were comparable to their baseline latencies. Cumulative dose-response curves of ENDO-2 were then constructed in both the ENDO-2 pre-treatment and Saline pre-treatment groups in the following manner. A single dose of ENDO-2 was injected, and the tail flick latency was taken at 2.5 min later (the peak analgesic time point). A second dose of ENDO-2 was delivered and the process repeated. Doses were delivered in this manner until a complete dose-response curve was constructed in each treatment group.

### 2.11 Intrathecal NMDA scratching and biting nocifensive behavioral assay

Intrathecal delivery of NMDA (0.3 nmol) results in biting and scratching of their hindlimbs that lasts for 1 min (39). These behaviors are counted and represent the dependent measure for this assay. Other agents intended to modify these behaviors may be co-administered with the NMDA or given as an intrathecal pre-treatment. In the present study, agmatine was given as a co-administration with NMDA. The anti-agmatine IgG was given as a five minute pre-treatment to the NMDA + agmatine combination.

### 2.12 Agmatine immunoneutralization

In these experiments, either normal guinea pig IgG (150 ng) or anti-agmatine IgG (150 ng) was injected intrathecally five minutes prior to the intrathecal injection of ENDO-2 (10 nmol), which was given to induce acute ENDO-2 tolerance. These IgGs were raised in-house and previously characterized and reported (20, 27, 31). Cumulative dose-response curves were performed 30 min after the ENDO-2 tolerance-inducing injection, as previously described in section 2.10.

## 3 Results

### 3.1 AAV5-hADC treatment prevents the acquisition of morphine analgesic tolerance

**Figure 1:**
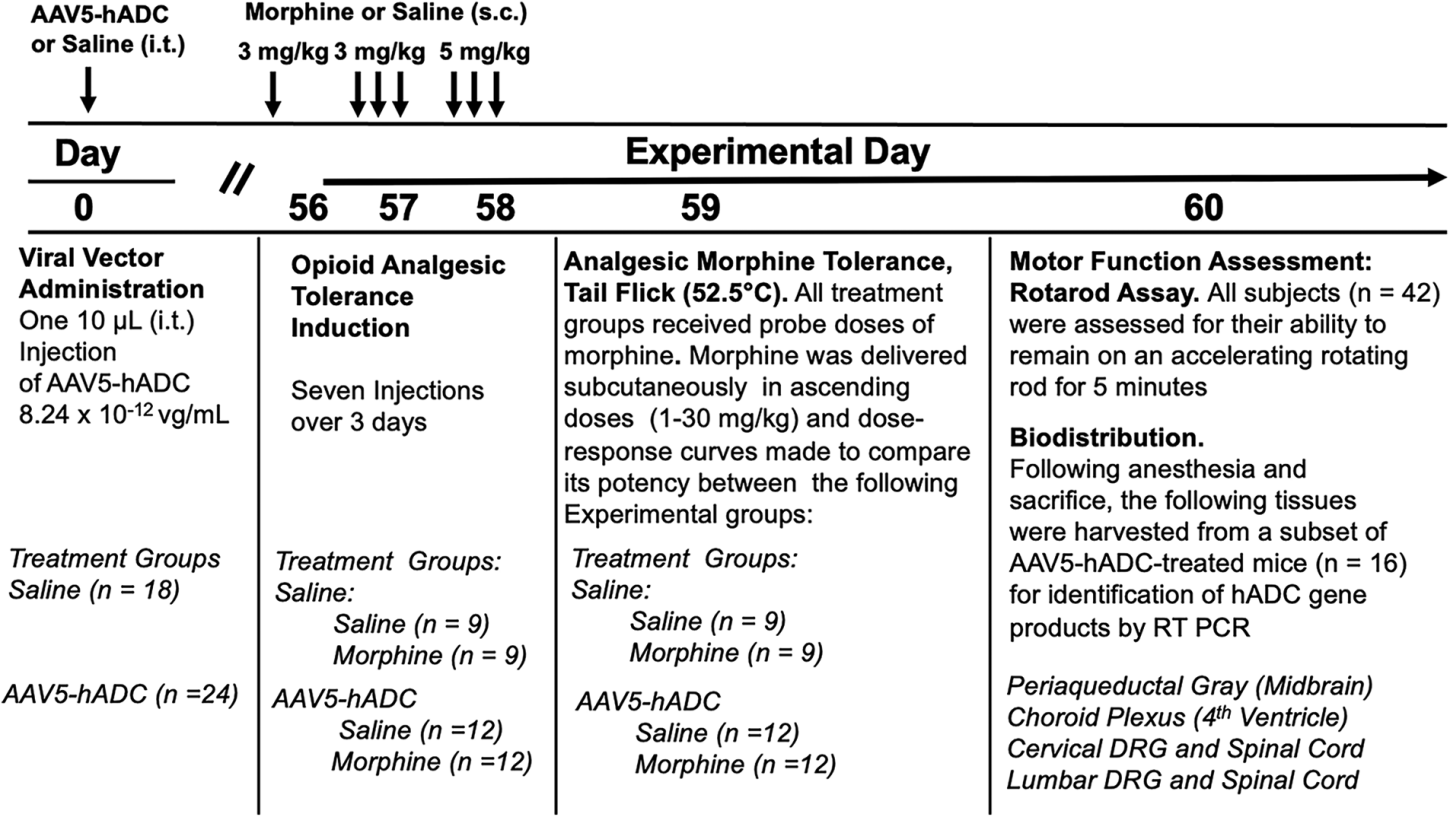
Study timeline: morphine tolerance in animals pre-treated with AAV5-hADC.

We proposed that expression of hADC in central nervous system reduces the acquisition of opioid analgesic tolerance. To test this hypothesis we compared the development of opioid analgesic tolerance in mice that had been injected before with either saline or AAV-hADC. The experimental plan is summarized in Figure 1.

Briefly, eight weeks after intrathecal delivery of saline or AAV5-hADC, the two groups were each divided into four experimental groups. Specifically, the saline-treated group and the AAV5-hADC-treated groups were each separated into two additional cohorts that received repeated doses of either morphine or saline over a 3 day period (Experimental Days 56–58, Figure 1). The following day (Experimental Day 59) all four subject groups were subcutaneously injected sequentially with increasing doses of probe morphine (1, 3, 10, 20 or 30 mg/kg.) mice. Probe dose-response curves for morphine were then constructed and the ED_50_ values and confidence intervals compared between the saline-injected and morphine-injected groups for each cohort (AAV5-hADC and vehicle pretreatment groups).

**Figure 2:**
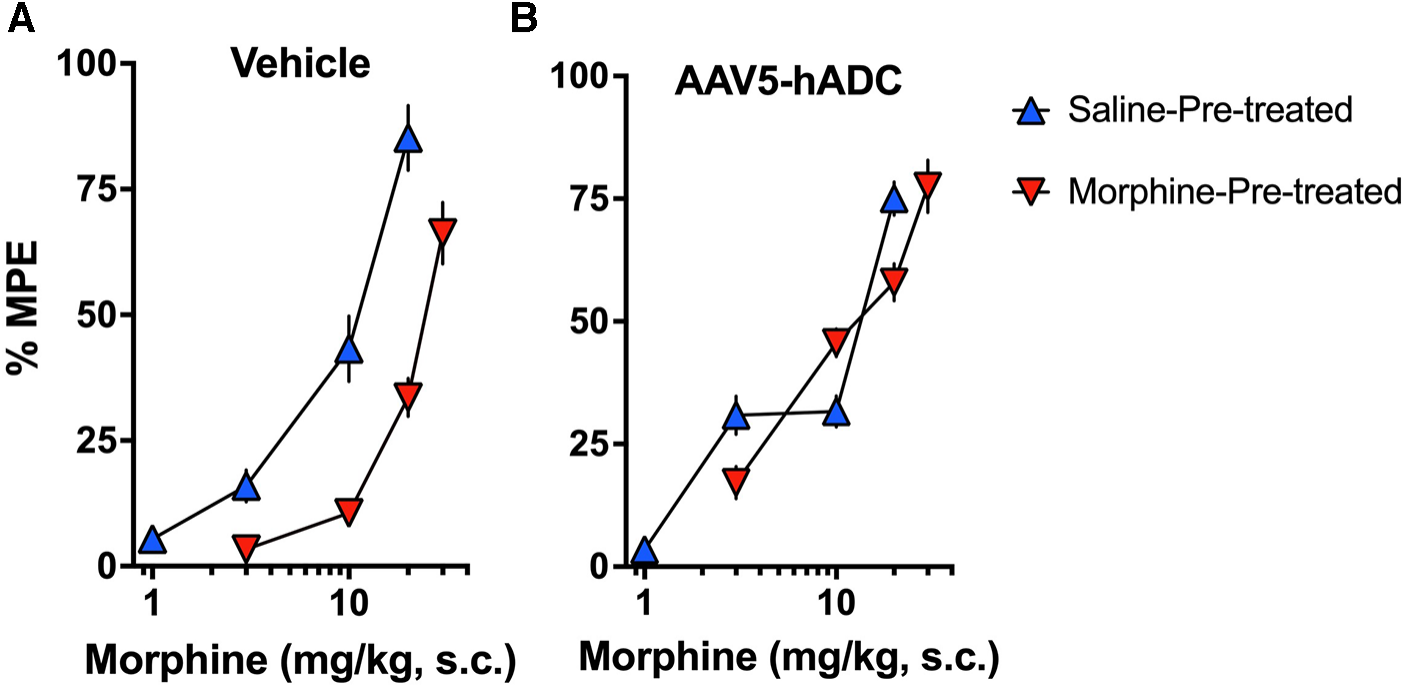
Effects of AAV5-hADC pre-treatment on acquisition of analgesic tolerance to morphine. Analgesic dose-response curves to acute subcutaneous (s.c.) probe morphine on Day 4 following repeated subcutaneous injection of either saline (blue triangles) or morphine (red inverted triangles). (**A**) Controls: Repeated s.c. injections of morphine (inverted red triangles) decreased acute probe morphine potency and efficacy compared with subjects repeatedly injected with saline (blue triangles), indicating induction of chronic morpine analgesic tolerance. (**B)** AAV5-hADC-treated subjects: Repeated s.c injections of morphine (inverted red triangles) demonstrated equivalent potency and efficacy compared with subjects repeatedly injected with saline (blue triangles), indicating that induction of chronic morpine analgesic tolerance did not occur in this experimental group.

As expected, repeated injections of s.c. morphine produced an approximate 3-fold rightward shift in the probe morphine dose-response curve compared to the probe morphine dose-response curve in the group that received repeated s.c. injections of saline (control group) in the cohort of mice that had previously received an intrathecal injection of saline (control to the AAV-hADC) (Figure 2A, Table 1). The rightward shift in the ED_50_ values confirms the induction of systemic morphine tolerance in control conditions. In contrast, when mice were pre-treated with intrathecal AAV5-hADC, repeated injections with s.c. morphine did not produce a shift in the probe morphine dose-response curve compared to those repeatedly injected with s.c. saline (Figure 2B). The ED_50_ values for morphine were equivalent between the two repeated injection groups (Table 1). The observation of no shift in the morphine dose-response curves indicates that morphine tolerance did not develop in subjects treated with AAV5-hADC.

**Table 1:** ED_50_ values for the dose-response curves in Figure 2.

Figure 2	8 week intrathecal Pre-treatments	Probe morphine ED_50_ Values (95% C.I.) (mg/kg s.c.)		Potency change
Panel	Treatment groups	Saline-treated	Morphine-treated	ED_50_ Mor-treated/ ED_50_ Sal-treated
A	Saline	8.9 (7.3–11)	24 (21–27)	2.7
B	AAV5-hADC)	10 (7.9–13)	12 (10–14)	1.2

### 3.2 AAV5-hADC treatment does not impact rotarod performance

**Figure 3:**
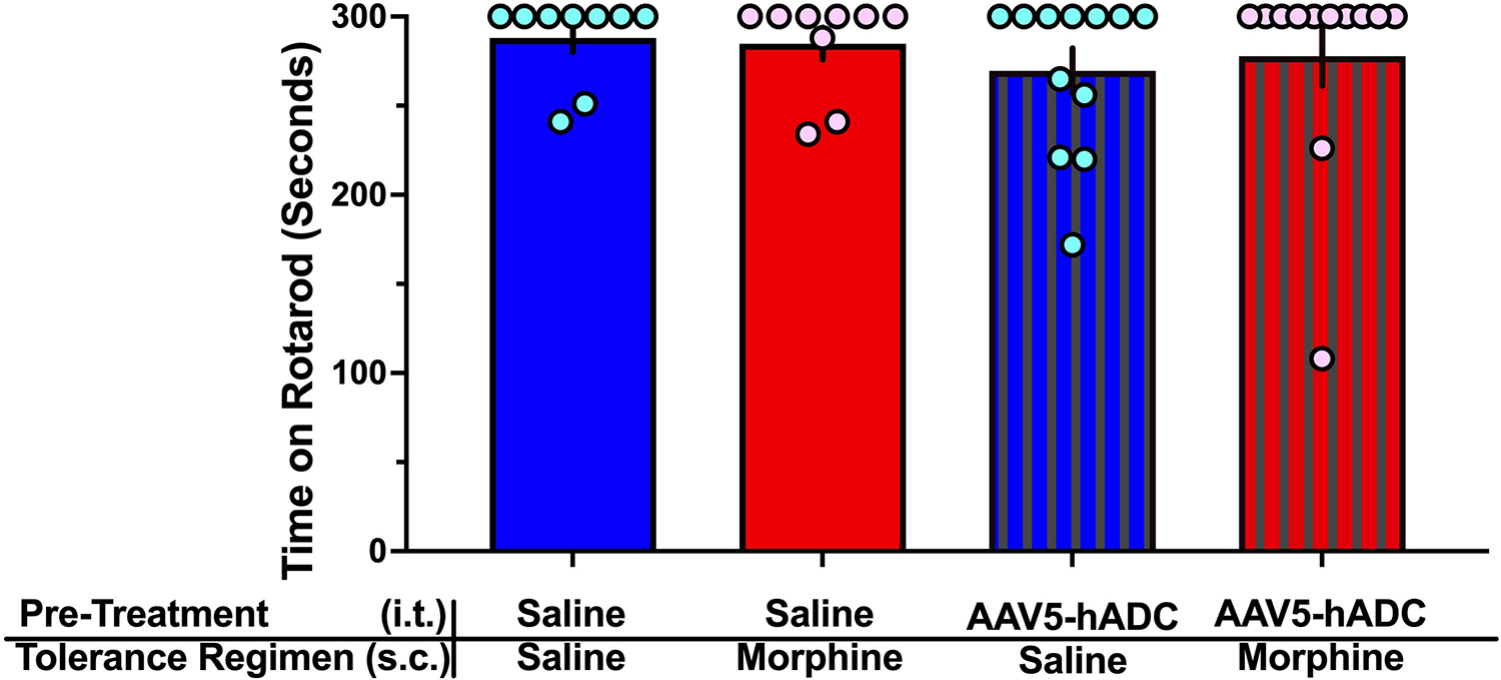
Rotarod assay of motor dysfunction/sedation. Comparison of rotarod performance in mice on Experimental Day 60 following treatment with (1) intrathecal saline and subcutaneous saline (blue bars, *n* = 9), (2) intrathecal saline and subcutaneous morphine (red bars, *n* = 9), (3) intrathecal AAV-hADC, and subcutaneous saline (blue striped bars, *n* = 12), and (4) intrathecal AAV-hADC and subcutaneous morphine (red striped bars, *n* = 12). There were no differences between groups as determined by a one-way ANOVA (*F*(3, 38) = 0.3967, *p* = 0.76) followed by Tukey's *post hoc* test for multiple comparisons between groups.

One day following completion of the morphine tolerance experiment, mice were assessed on the rotarod for any motor deficits. None was noted in any treatment group (Figure 3).

### 3.3 AAV5-hADC treatment results in hADC expression in the sensory system

Rodents express a form of ADC that is distinct from that of the human sequence. Since a human form of ADC was introduced, we are able to distinguish hADC from native mouse ADC (27). Therefore following conclusion of the rotarod experiments, subjects were sacrified and various tissues extracted and processed for presence of hADC mRNA by real-time PCR. hADC mRNA was detected in lumbar and cervical spinal cord and dorsal root ganglion tissue, choroid plexus (4th ventricle) and periaqueductal gray. The tissue distribution and the percentages of AAV5-hADC expressed in these tissues are summarized in Table 2.

**Table 2:** AAV5-hADC: percentage of subjects expressing hADC mRNA in diverse tissues.

Tissue region	Periaqueductal gray (PAG)	Choroid plexus (4th Ventricle)	Cervical DRG	Cervical spinal cord	Lumbar DRG	Lumbar spinal cord
Post-AAV5-hADC injection (*n* = 16)	50% (8 of 16)	87% (13 of 15)	75% (12 of 16)	81% (13 of 16)	81% (13 of 16)	88% (14 of 16)

### 3.4 AAV9-hADC treatment prevents the acquisition of morphine analgesic tolerance

**Figure 4:**
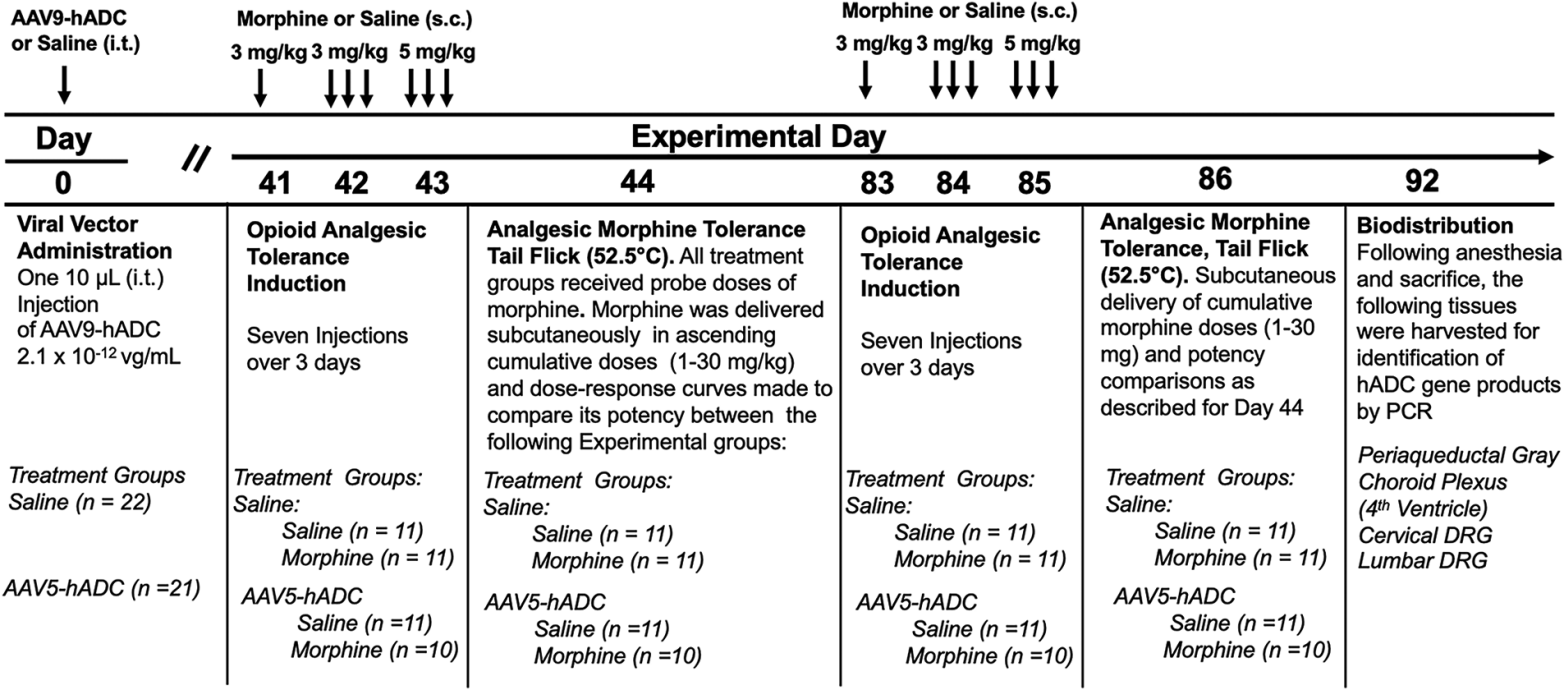
Study timeline: morphine tolerance in animals treated with AAV9-hADC.

To determine whether the prevention of opioid tolerance in AAV-hADC-treated mice was generalizable to a second AAV serotype, we performed a similar experiment using an AAV9-hADC vector. In this case, we tested the subjects for opioid analgesic tolerance at approximately six weeks after AAV9-hADC treatment and then repeated the experiemnt at approximately 12 weeks post treatment. The general experimental design is as featured in Figure 4.

Briefly, six weeks following intrathecal delivery of either saline or AAV9-hADC, the two groups were each divided further into four experimental groups as described in Figure 4. The saline-treated group and the AAV9-hADC-treated groups were each separated into two additional groups that received repeated doses of either morphine or saline over a 3 day period (Experimental Days 41–43). The following day (Experimental Day 44) all four subject groups were subcutaneously injected sequentially with increasing doses of probe morphine (1, 3, 10, 20 or 30 mg/kg.) mice. Probe morphine dose-response curves were then constructed and compared between the saline and morphine-injected groups for each cohort (AAV9-hADC and saline pretreatment groups).

**Figure 5:**
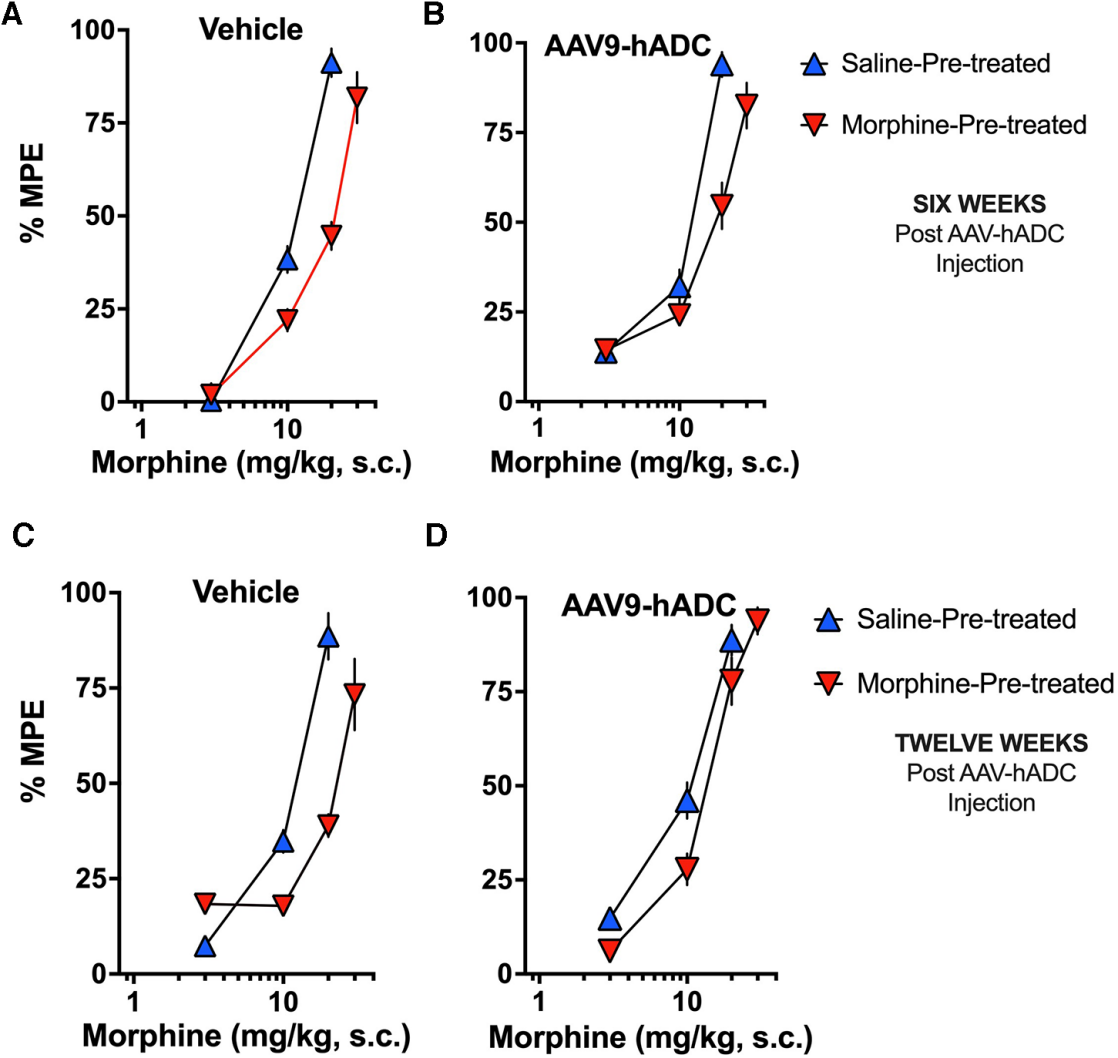
Effects of AAV9-hADC pre-treatment on development of morphine tolerance. Analgesic dose-response curves to acute subcutaneous (s.c.) probe morphine on Day 4 following repeated subcutaneous injection of either saline (blue triangles) or morphine (inverted red triangles). Six Weeks Post-Vehicle (Saline) or AAV9-hADC I.T. injection. (**A**) Controls: Repeated s.c. injections of morphine (inverted red triangles) decreased acute probe morphine potency and efficacy compared with subjects repeatedly injected with saline (blue triangles), indicating induction of chronic morphine analgesic tolerance. (**B**) AAV9-hADC-treated subjects: repeated s.c injections of morphine (inverted red triangles) decreased acute probe morphine potency compared with subjects repeatedly injected with saline (blue triangles), indicating induction of chronic morphine analgesic tolerance. (**C**, **D**) Twelve Weeks Post-Vehicle (Saline) or AAV9-hADC I.T. injection. Controls: Repeated s.c. injections of morphine (inverted red triangles) decreased acute probe morphine potency and efficacy compared with subjects repeatedly injected with saline (blue triangles), indicating induction of chronic morphine analgesic tolerance. (**D**) AAV9-hADC-treated subjects: repeated s.c injections of morphine (inverted red triangles) demonstrated equivalent potency and efficacy compared with subjects repeated injected with saline (blue triangles), indicating that induction of chronic morphine analgesic tolerance did not occur in this experimental group.

In the intrathecal saline pre-treatment group, repeated injections of s.c. morphine produced a 1.7-fold rightward shift in the probe morphine dose-response curve (Figure 5A) relative to the probe morphine dose-response curve in the group that received repeated s.c. saline (control group). Repeated morphine injections increased the acute morphine ED_50_ value (Table 3). Similarly, when mice were pre-treated with intrathecal AAV9-hADC, repeated injections with morphine produced a 1.5-fold shift in the acute probe morphine dose-response curve when compared to those repeatedly injected with s.c. saline (Figure 5B). In this experiment, repeated morphine injections increased the acute morphine ED_50_ value in the AAV9-hADC-treated group to nearly the same magnitude as the control group (Table 3). We speculate that the six week pre-treatment was insufficient for AAV9-hADC delivered at a titer of 2.1 × 10^12^ vector genomes/ml to manifest the effect. Therefore, we re-tested the same subjects at 12 weeks post-injection in the same morphine analgesic tolerance induction schedule (Figures 5C,D).

**Table 3:** ED_50_ values for the dose-response curves in Figure 5.

Figure 5	Treatment groups	Probe morphine ED_50_ Values (95% C.I.) (mg/kg s.c.)		Potency change
Panel		Saline-treated	Morphine-treated	ED_50_ mor-treated/ED_50_ sal-treated
6 week intrathecal pre-treatment				
A	Saline	9.8 (8.8–11)	17 (14–21)	1.7
B	AAV9-hADC	9.2 (7.6–11)	14 (12–18)	1.5
12 week intrathecal Pre-treatments				
C	Saline	9.9 (8.5–12)	21 (18–24)	2.1
D	AAV9-hADC	8.5 (7.4–9.6)	11 (9.8–13)	1.3

In the control mice, repeated injections of s.c. morphine produced a 2.1-fold rightward shift in the probe morphine dose-response curve relative to the probe morphine dose-response curve in the group that received repeated s.c. saline (Figure 5C). Repeated morphine injections doubled the acute morphine ED_50_ value (Table 3). This result demonstrates that morphine tolerance developed in the control group, as expected. In contrast, repeated injections with morphine produced a 1.3-fold shift in the probe morphine dose-response curve compared to those repeatedly injected with s.c. saline in the mice pre-treated with AAV9-hADC (Figure 5D, Table 3). The observation of no shift in the morphine dose-response curve indicates that morphine tolerance did not develop in subjects treated with AAV9-hADC, similar to what was observed in the AAV5-hADC treated subjects represented in Figure 2.

As in Figure 2, the subjects were sacrificed and various tissues extracted and processed for presence of hADC mRNA by real-time PCR. hADC mRNA was detected in cervical and lumbar dorsal root ganglion tissue and choroid plexus (4th ventricle). The tissue distribution and the percentages of AAV9-hADC expressed in these tissues are summarized in Table 4.

**Table 4:** AAV9-hADC: percentage of subjects expressing hADC mRNA in diverse tissues.

Tissue region	Periaqueductal gray (PAG)	Choroid plexus (4thVentricle)	Cervical DRG	Lumbar DRG
Post-AAV9-hADC injection (*n* = 20)	0% (0 of 20)	20% (4 of 20)	25% (5 of 20)	70–75% (14 or 15 of 20)^a^

^a^ 2 samples were inadvertently loaded into the same tube and were not distinguishable. The outcome was positive. Therefore, it was either 14 or 15 of 20 samples that were positive.

### 3.5 Agmatine immunoneutralization prevents AAV5-hADC-mediated inhibition of opioid analgesic tolerance

**Figure 6:**
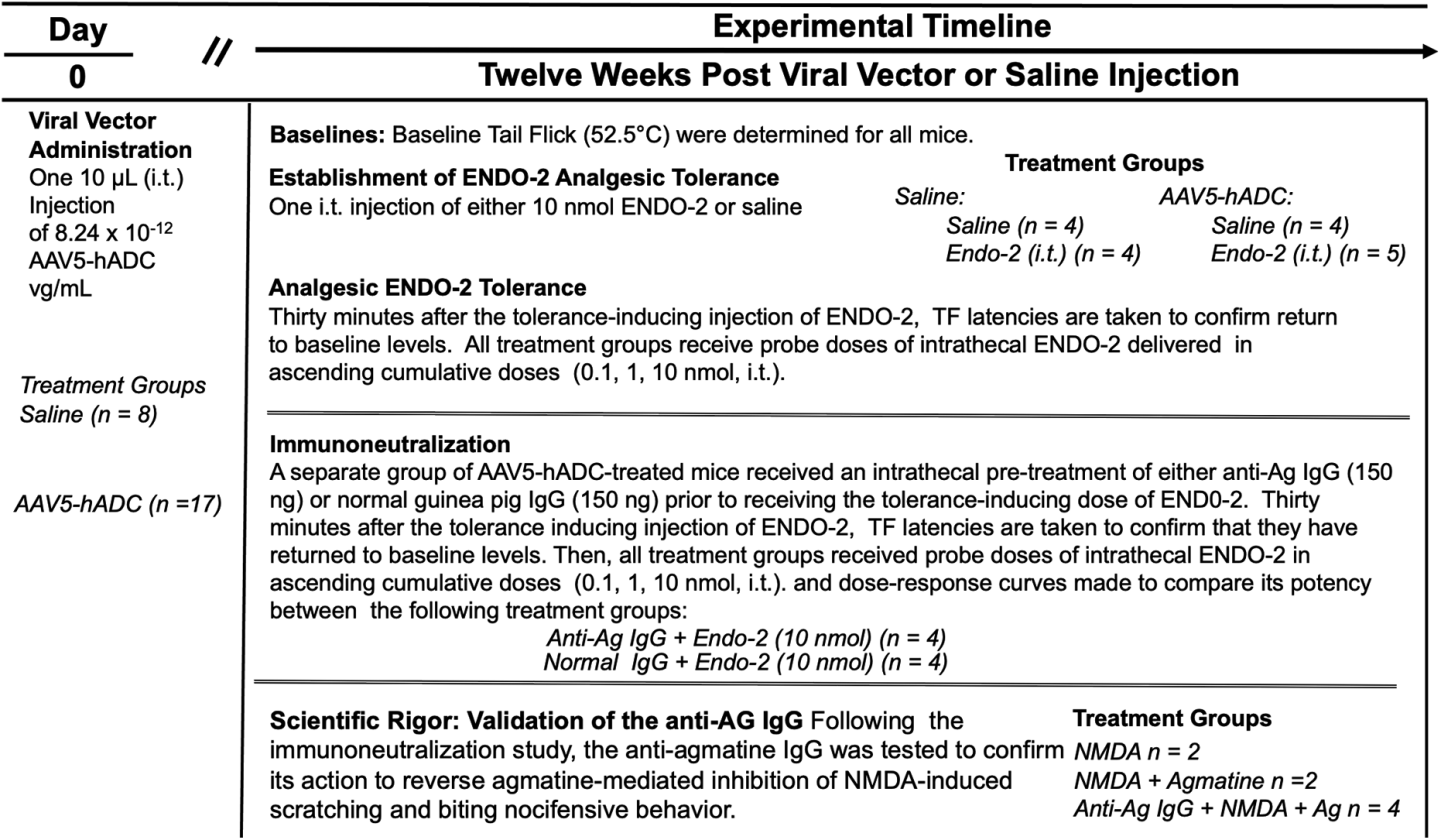
Study timeline: ENDO-2 tolerance in animals treated with AAV5-hADC.

We used an immunoneutralization strategy to determine whether the prevention of opioid tolerance in AAV5-hADC-treated mice was due to an effect of the intended product, agmatine. The impact of intrathecal delivery of an immunoneutralizing antibody (anti-Ag IgG) was assessed in a model of opioid-induced analgesic tolerance as previously described (20). For this experiment, the opioid neuropeptide, endomorphin-2 (ENDO-2) (YPFF) (40), was used as the analgesic tolerance-inducing agent. It was previously demonstrated (20, 41) that mice given higher intrathecal doses of ENDO-2 (10–30 nmol, i.t.) develop acutely induced analgesic tolerance. It was also shown previously that intrathecal pre-treatment of an antibody raised to selectively target agmatine (20, 31) (anti-Ag IgG) sensitizes mice to the development of ENDO-2 analgesic tolerance induced by low doses (20). We used the ENDO-2 model of acute opioid tolerance to test for the effect of agmatine immunoneutralization in the hADC-treated mice. The overall experimental design is summarized in Figure 6.

Approximately twelve weeks following intrathecal delivery of either saline or AAV5-hADC, the two groups were each divided into four experimental groups. The saline-treated group and the AAV5-hADC-treated groups were each divided into two additional groups that received a single intrathecal injection of either ENDO-2 (10 nmol) or saline. ENDO-2 has a very short duration of action (41). Thirty minutes after the initial high dose injection, mice were tested cumulatively with increasing doses of ENDO-2. The tail flick test was performed 2.5 min after the test injection. All four subject groups were intrathecally injected sequentially with increasing doses of probe ENDO-2 (1, 3, 10, or 20 nmol). Probe ENDO-2 dose-response curves were then constructed and the ED_50_ values and confidence intervals compared between the saline-injected and ENDO-2-injected groups for each cohort (AAV5-hADC and vehicle pretreatment groups).

**Figure 7:**
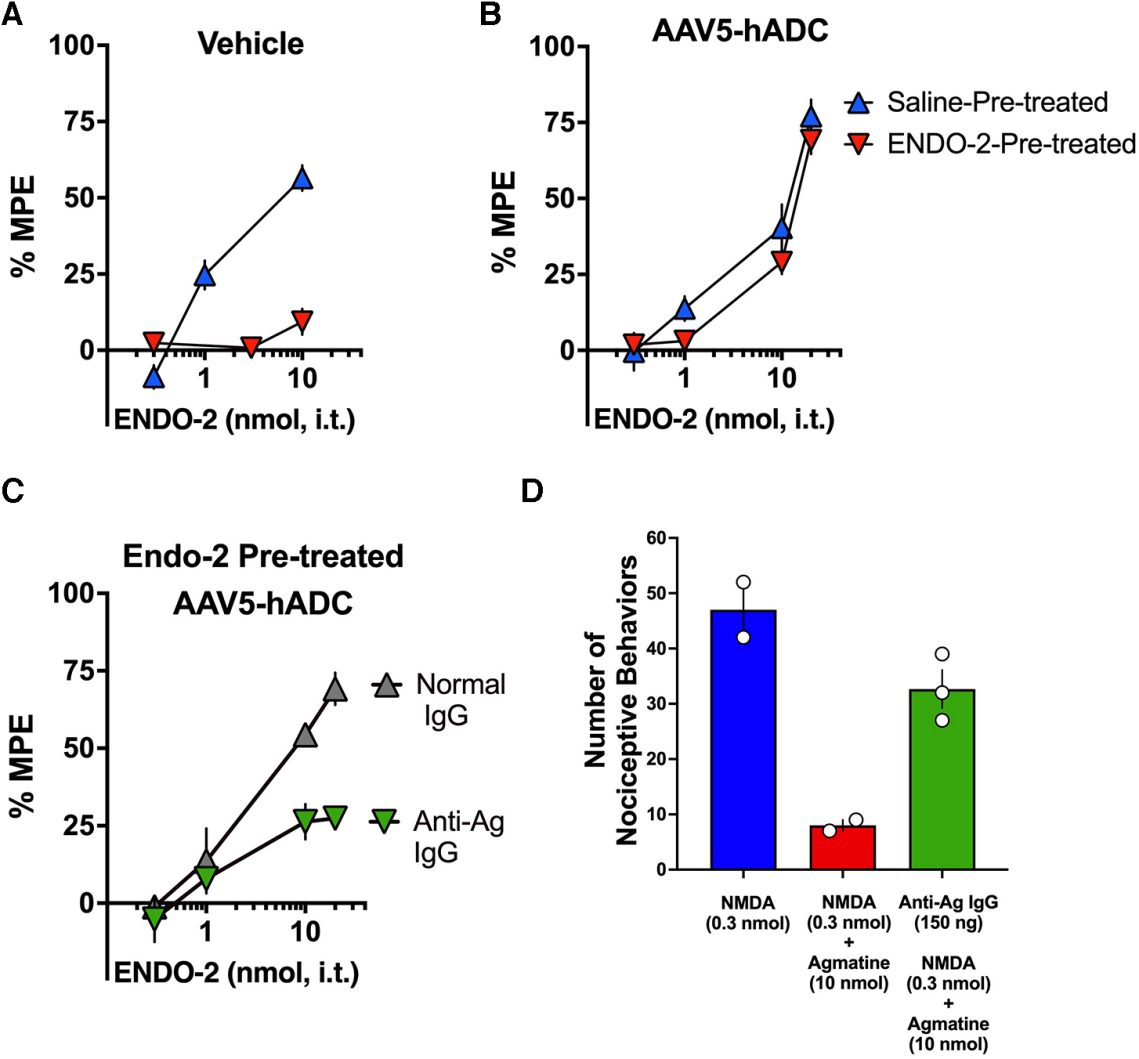
Effects of AAV5-hADC pre-treatment on the development of ENDO-2 tolerance. Analgesic dose-response curves to acute intrathecal (i.t.) probe ENDO-2 30 min following a single acute injection of either saline (blue triangles) or ENDO-2 (red inverted triangles). (**A**) *Vehicle-Treated Controls*: a single acute injection of ENDO-2 (inverted red triangles) decreased acute probe ENDO-2 potency compared with subjects injected with saline (blue triangles), indicating induction of acute ENDO-2 analgesic tolerance. (**B**) *AAV5-hADC-treated subjects* that received a single i.t. injection of ENDO-2 (inverted red triangles) demonstrated equivalent potency compared with AAV5-hADC-treated subjects subjects injected with saline (blue triangles), indicating that induction of acute ENDO-2 analgesic tolerance did not occur when subjects where pre-treated with AAV5-hADC. (**C**) AAV5-hADC-treated subjects injected with tolerance-inducing dose of ENDO-2. Subjects that received a pre-treatment with normal IgG (grey triangles) demonstrated equivalent potency and efficacy compared with AAV5-hADC-treated that received a tolerance-inducing dose of ENDO-2 (Panel B, red inverted triangles). Subjects that received a pre-treatment with anti-agmatine IgG (inverted green triangles) demonstrated greatly reduced probe ENDO-2 efficacy. (**D**) NMDA (0.3 nmol i.t) induces nociceptive behaviors (blue bar), which is reduced with co-injection with agmatine (10 nmol, red bar). Pre-treatment with anti-agmatine IgG attenuates the effect of agmatine (green bar).

In control mice that had received a single i.t. injection of saline (as controls to AAV5-hADC), probe doses of ENDO-2 produced dose-dependent analgesia in the tail flick test in mice that received a single 30 min i.t. pre-treatment with saline. However, probe ENDO-2 doses failed to produce analgesia in control mice that had previously received a single high dose injections of i.t. ENDO-2 (Figure 7A). These data confirm the induction of acute ENDO-2 tolerance in control conditions. In contrast, when mice were pre-treated with intrathecal AAV5-hADC, a single high dose injection with i.t. ENDO-2 did not produce a reduction in either efficacy or potency in the probe ENDO-2 dose-response curve compared to those that received a single i.t. dose of saline (Figure 7B). The ED_50_ values for ENDO-2 were equivalent between the two injection groups (Table 5). The observation of no shift in the ENDO-2 dose-response curves or no reduction in efficacy indicates that ENDO-2 tolerance did not develop in subjects treated with AAV5-hADC.

**Table 5:** ED_50_ values for the dose-response curves in Figure 7.

Figure 7	12 week intrathecal Pre-treatments	Probe Endo-2 ED_50_ Values (95% C.I.) (mg/kg s.c.)		Potency Change
Panel	Treatment groups	Saline-treated	ENDO-2-treated	ED_50_ ENDO-2-treated/ED_50_ sal-treated
A	Saline	6.0 (3.7–10)	Not Calculable	N/A
B	AAV5-hADC	7.9 (4.5–14)	15 (7.6–29)	1.9

We used an immunoneutralization strategy to determine whether the prevention of ENDO-2 acute tolerance by the AAV5-hADC pre-treatment was due to an effect of agmatine. We tested the impact of intrathecal delivery of an anti-agmatine IgG on the prevention of opioid tolerance observed in AAV-hADC-treated mice. We have previously shown that intrathecal delivery of anti-agmatine IgG dose-dependently reverses agmatine-mediated inhibition of scratching and biting nociceptive behaviors induced by intrathecal delivery of NMDA (20). To perform the immunoneutralization experiment, we compared the analgesic efficacy of probe ENDO-2 in AAV5-hADC-injected mice that had all received a high dose of ENDO-2 but that had also received an intrathecal pre-treatment of either anti-agmatine IgG or control normal IgGs.

One group of AAV5-hADC-treated mice received an intrathecal injection of normal guinea pig IgG (150 ng) prior to the high dose of ENDO-2 that is used to induce acute tolerance. Similar to the result of Figure 7B, the probe analgesic potency of ENDO-2 in these mice was not reduced. These data suggest that the AAV5-hADC pretreatment protected against the acquisition of ENDO-2 analgesic tolerance. In contrast, the probe analgesic potency of ENDO-2 in AAV-hADC-treated mice that received a pre-treatment of anti-agmatine IgG was greatly reduced (Figure 7C). As a point of scientific rigor, we tested the anti-Ag IgG used in Figure 7C for reversal of agmatine-mediated inhibition of NMDA-evoked biting and scratching behaviors. NMDA (0.3 nmol) elicited biting and scratching nociceptive behaviors which were attenuated with co-administration with agmatine (10 nmol). In mice pre-treated with anti-agmatine IgG (150 ng), the inhibitory effect of agmatine was greatly diminished (Figure 7D). This outcome verified the integrity of the anti-agmatine IgG aliquot that was used for the immunoneutralization experiment. We interpret these findings to mean that the anti-agmatine IgG immunoneutralized the protective effect of the AAV5-hADC pretreatment in the development of ENDO-2 analgesic tolerance, presumably through rendering elevating agmatine unavailable to exert its effects.

## 4 Discussion

We have recently demonstrated that intrathecal delivery of an AAV vector containing the gene for human arginine decarboxylase (hADC) persistently reduces neuropathic pain in mice (27). The present study reveals that intrathecal delivery of the same AAV vector containing the hADC gene also prevents the acquisition of analgesic tolerance to opioids in mice. It is noteworthy that the effect was observed with two distinct opioid agonists (morphine and endomorphin-2) and with two distinct AAV serotypes.

Both neuropathic pain and opioid analgesic tolerance share a well-established dependence on the NMDA receptor/NOS cascade (30). The presumptive product of hADC, decarboxylated L-arginine or agmatine, is a known antagonist of the NMDA receptor (22, 42) and the NOS enzyme (23). We have previously shown that agmatine reduces neuropathic pain (27, 31, 32) and prevents the development of opioid analgesic tolerance (18–20). We have also demonstrated that agmatine reduces spinal long-term potentiation evoked by high frequency stimulation (27). The present observation of reduction of opioid tolerance by gene transfer of hADC is consistent with the prior reported pharmacology of agmatine.

To determine whether endogenous agmatine accounted for the prevention of opioid tolerance observed in subjects treated with the AAV-hADC, we used an immunoneutralization approach. We observed that pre-treatment with an anti-agmatine IgG (but not normal IgG) reversed the anti-opioid analgesic tolerance effect observed in AAV-hADC-treated mice. Immunoneutralization strategies using scavenging antisera have been applied extensively to characterize the actions of endogenous analgesic substances (43–45) and recently endogenous pro-nociceptive substances (46). A recent study also used an immunoneutralization approach to evaluate pharmacological actions of a presumptive endogenous NMDA receptor antagonist, serine histogranin, arising from intraspinal delivery of an AAV2/AAV8 vector carrying its synthetic gene (47). We previously demonstrated that the structure-specific anti-agmatine immunoglobulin G (anti-Ag IgG) dose-dependently reversed the pharmacological effects of exogenously applied intrathecal agmatine in the NMDA test of nocifensive scratching and biting behavior (20). Therefore, we theorized that intrathecal delivery of the anti-Ag IgG antibody would prevent the pharmacological activity of agmatine presumed to be generated by the expression of hADC. In our previous report (27) we demonstrated that hADC gene transfer reduced tactile hypersensitivity arising from nerve injury. We demonstrated that intrathecal anti-Ag IgG (but not normal IgG) transiently reversed the alleviation of mechanical allodynia observed in AAV5-hADC-treated mice. We also observed that anti-Ag IgG potentiated the magnitude of spinal long-term potentiation induced in rat by low frequency stimulation (27), presumably due to reducing the concentration of spinal agmatine available to moderate excitation. Consistent with this observation, in this present study, intrathecally delivered anti-agmatine IgG (but not normal IgG) increased the magnitude of ENDO-2 analgesic tolerance induced by high dose spinal ENDO-2. These observations support the proposal that elevated endogenous agmatine contributes to the anti-neuroplasticity effects arising from gene transfer associated with the AAV5-hADC treatment.

It is notable that agmatine robustly inhibits the development of opioid-induced tolerance (17–19), but without motor toxicity (31, 48), which is commonly associated with NMDA receptor antagonists, such as MK801. Consistent with those prior findings, AAV5-hADC-treated mice did not demonstrate altered performance in rotarod compared to control subjects at the time point assessed.

In our previous report (27) we detected hADC mRNA expression in the sensory system (e.g., spinal cord and dorsal root ganglia) and supraspinal regions (choroid plexus) following intrathecal delivery of AAV-hADC vectors. In the present study we similarly detected hADC mRNA in dorsal root ganglia, spinal cord and choroid plexus, but also in periaqueductal gray (with the hADC-AAV5 vector), a region important to opioid action. Prior studies of the biodistribution of green fluorescent protein immunoreactivity in spinal cord and DRG (29, 49, 50) suggest that the AAV5 and AAV9 serotypes used in these experiments differentially target sensory neurons. Additionally, following intrathecal delivery of either AAV5 (49) or AAV9 (50), we have observed expression of GFP in various regions of the brain, including the choroid plexus, presumably from rostral diffusion of the viral particles (49). Therefore, the mRNA expression data presented here are consistent with the prior biodistribution studies using the GFP marker following intrathecal delivery of AAV5-GFP and AAV9-GFP. We speculate that hADC expressed in sensory neurons terminating in the dorsal horn of the spinal cord elevated agmatine production that exerted an effect similar to exogenously injected agmatine in models of morphine tolerance (18). Additionally, we further speculate that the expression of hADC in epithelial cells of the choroid plexus contributed to the elevation of agmatine via release of agmatine into the cerebrospinal fluid (CSF). This could cause a broad distribution of the molecule throughout the CNS that could act in a manner comparable to that observed in prior pharmacological studies demonstrating efficacy of i.c.v.-injected agmatine in blocking morphine analgesic tolerance (19).

It has been established that co-administration of NMDA receptor antagonists with opioids prevents the development of opioid tolerance (6). This strategy has been used clinically to “reset” a patient's opioid analgesic efficacy and potency when opioid analgesic tolerance has arisen (51, 52). However, initial efforts to develop a combined preparation of opioid + NMDA receptor antagonist have not yet advanced to practice (54). As stated previously, what distinguishes agmatine from other NMDA receptor antagonists/NOS inhibitors is that it is endogenous and, therefore, may present a highly localized and effective gene therapy to counter maladaptive neuroplasticity. The present study features an application of gene transfer to sensory neurons, the midbrain, and choroid plexus epithelial cells using direct lumbar puncture. As gene therapeutics are further developed, optimized, and translated into clinical applications, intrathecal gene therapy as an adjuvant to chronic opioid pharmacotherapy may become more broadly recognized as a potentially effective pain management strategy.

## Data Availability

The raw data supporting the conclusions of this article will be made available by the authors, without undue reservation.
